# Disentangling self-management goal setting and action planning: A scoping review

**DOI:** 10.1371/journal.pone.0188822

**Published:** 2017-11-27

**Authors:** Stephanie Anna Lenzen, Ramon Daniëls, Marloes Amantia van Bokhoven, Trudy van der Weijden, Anna Beurskens

**Affiliations:** 1 Research Centre for Autonomy and Participation for People with a Chronic Illness, Zuyd University of Applied Sciences, Heerlen, the Netherlands; 2 Department of Family Medicine, CAPHRI School for Public Health and Primary Care, Maastricht University, Maastricht, the Netherlands; University of Quebec at Montreal, CANADA

## Abstract

**Introduction:**

The ongoing rise in the numbers of chronically ill people necessitates efforts for effective self-management. Goal setting and action planning are frequently used, as they are thought to support patients in changing their behavior. However, it remains unclear how goal setting and action planning in the context of self-management are defined in the scientific literature. This study aimed to achieve a better understanding of the various definitions used.

**Methods:**

A scoping review was conducted, searching PubMed, Cinahl, PsychINFO and Cochrane. Inclusion and exclusion criteria were formulated to ensure the focus on goal setting/action planning and self-management. The literature was updated to December 2015; data selection and charting was done by two reviewers. A qualitative content analysis approach was used.

**Results:**

Out of 9115 retrieved articles, 58 met the inclusion criteria. We created an overview of goal setting phases that were applied (preparation, formulation of goals, formulation of action plan, coping planning and follow-up). Although the phases we found are in accordance with commonly known frameworks for goal setting, it was striking that the majority of studies (n = 39, 67%) did not include all phases. We also prepared an overview of components and strategies for each goal setting phase. Interestingly, few strategies were found for the communication between patients and professionals about goals/action plans. Most studies (n = 35, 60%) focused goal setting on one single disease and on a predefined lifestyle behavior; nearly half of the articles (n = 27, 47%) reported a theoretical framework.

**Discussion:**

The results might provide practical support for developers of interventions. Moreover, our results might encourage professionals to become more aware of the phases of the goal setting process and of strategies emphasizing on patient reflection. However, more research might be useful to examine strategies to facilitate communication about goals/action plans. It might also be worthwhile to develop and evaluate goal setting/action planning strategies for people with different and multiple chronic conditions.

## Introduction

The increasing numbers of people living with one or more chronic conditions worldwide necessitate efforts to achieve effective self-management [1]. Based on the Expanded Chronic Care Model, self-management is defined as the degree to which a patient with a chronic condition is able to, and wants to, control his own daily life, by coping effectively with symptoms, treatments and lifestyle adjustments [2, 3]. Self-management support is defined as the support provided by healthcare professionals to enable patients to become good managers of their chronic conditions, tailored to their needs and capabilities [2, 4]. The aim of successful self-management is to enhance patient autonomy, to improve health status and consequently reduce the use of health services, leading to a decrease of the financial pressure on the healthcare sector [5, 6].

Self-management includes more than just coping with medical symptoms of a chronic illness and compliance with medical treatment. In addition to medical self-management, Corbin & Strauss (1988) describe two other forms: social and emotional self-management [7]. Social management refers to the ability of a patient to adjust his or her behavior so as to prevent negative outcomes and maintain or adjust life roles. Emotional self-management refers to dealing with emotional responses to a chronic condition, such as fear and depression, and coping with discomfort and disability [7].

Self-management support begins with the exploration of patients’ beliefs and values [8]. Depending on what is important to the patient, goals for the patient’s self-management can be set and actions can be planned [8]. Within the primary care sector, the term ‘collaborative goal setting’, defined as a process in which healthcare professionals and patients agree on health-related goals, is frequently used [9]. Action planning is defined as agreeing on a course of action for the patient (and/or the professional), including questions like what, when, where and how often [9, 10]. Goal setting and action planning are frequently applied in self-management support programs, as they are found to improve patients’ self-efficacy, help them change their behavior and improve their health outcomes [5, 10–12]. Moreover, there is increasing attention on coping planning, in close association with goal-setting and action planning [9, 13]. Coping planning is defined as the formulation of plans to overcome potential barriers to carrying out an action plan [13, 14]. Nowadays, European treatment guidelines for chronic conditions, like diabetes or COPD, recommend goal setting and action planning as methods for self-management support [15, 16]. However, professionals frequently encounter difficulties in the day-to-day practice of goal setting and action planning practice [17]. The process is perceived as time-consuming and the effort involved and the complexity are often underestimated [18, 19].

Literature about goal setting has mostly focused on the experiences and skills of professionals and patients [20–23]. In addition, two reviews of the evidence on goal setting and action planning have become available, focusing on the rehabilitation context and assessing the effects of goal setting and action planning on patients’ health outcomes [21, 24]. However, information about goal setting and action planning in the context of self-management is often lacking. A more thorough understanding of goal setting and action planning might provide practical support to those developing self-management programs. We therefore conducted a scoping review and formulated the following research question: How is goal setting and action planning within the context of self-management defined in the scientific literature?

## Methods

### Scoping review

For this scoping review, we adopted the methodological framework developed by Arksey & O’Malley (2005) [25]. We chose this approach as it allows for the inclusion of many different study designs which fits our aim to give a broad overview of the way researchers define goal setting and action planning [25]. In contrast to systematic reviews, the quality of evidence is not evaluated in a scoping review. Instead, it addresses broader topics and research questions [25]. Arksey & O’Malley suggest that there are five stages to a scoping review: (1) identifying the research question; (2) identifying relevant studies; (3) selecting studies; (4) charting the data; and (5) collating, summarizing and reporting the results [25].

### Identifying relevant studies

The search strategy consisted of the following formula: goal setting/action planning AND method AND self-management. Search terms for ‘goal setting’ included the MeSH terms ‘Goals’ and ‘Patient Care Planning’, combined with free-text terms such as ‘goal setting’ and ‘goal planning’. Search terms for ‘method’ included the MeSH terms ‘Professional Practice’, ‘Technology’ and ‘Instrumentation’, combined with free text terms like ‘method*’ and ‘intervention*’. Finally, search terms for ‘self-management’ included the MeSH terms ‘Self Care’, ‘Disease Management’, ‘Patient Education’, ‘Patient Participation’, ‘Self-Help Groups’, ‘Personal Autonomy’, ‘Patient-Centered Care’ and ‘Patient Preference’ combined with free-text terms like ‘self-management support’ and ‘self-control’ (see Table 1 Search Strategy.).

**Table 1:** Search strategy.

	Goal setting/Action planning	Method	Self-management
**MeSH terms**	Goals Patient Care Planning	Professional practice Technology Instrumentation	Self-Care Disease management Patient Education Patient Participation Self Help group Personal Autonomy Patient-Centered care Patient Preference
**Free-text terms**	goal setting goal plan* action plan* coping plan* shared decision making	tool* method* process* procedure* framework* practice* intervention* technolog* instrument*	self-monitoring self-management self-control self-regulation self-help self-determination patient autonomy self-management support client cent* patient cent* empowerment

The PubMed, Cinahl, PsychINFO and Cochrane databases were searched during September 2013 with no restriction on the date of publication. In addition, reference lists of relevant articles were screened to identify key articles that had been missed. The literature was subsequently updated to December 2015.

### Selecting studies

The search was limited to adults (aged 19 years or older), English, Dutch, French and German articles and research involving humans. Study selection took place in three stages: first, titles were reviewed, followed by a review of abstracts and then full texts. Articles without an available abstract were directly included in the full-text review stage. Two researchers reviewed the articles. At each stage, a selection of 200 articles was reviewed by both reviewers to reach consensus about applying the inclusion and exclusion criteria. After that, each reviewer reviewed the articles independently.

**Fig 1:**
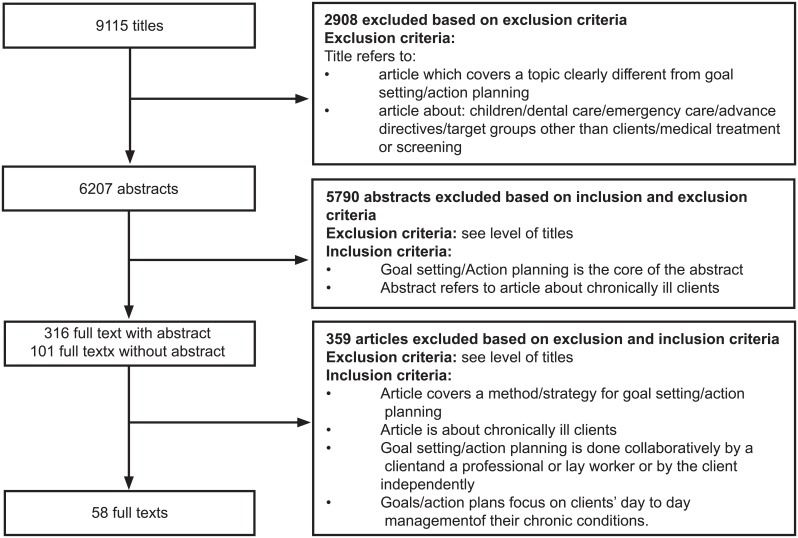
Study selection.

We formulated inclusion and exclusion criteria. Articles were included when: (a) the article focused on chronically ill patients, and (b) the terms ‘goal setting’ and/or ‘action planning’ or relevant synonyms were included, along with a definition of goal setting and/or action planning. To ensure that the articles defined goal setting and/or action planning within the context of self-management in the articles, additional inclusion criteria were formulated, building upon the self-management framework by Lorig & Holman (2003) [5]. The following criteria had to be met: (a) goal setting/action planning is done collaboratively by a patient and a professional or lay worker or independently by the patient, and (b) goals/action plans focus on patients’ day-to-day management of their chronic conditions. Articles were excluded when they focused on: (a) children/adolescents; (b) dental care; (c) emergency care, and/or (d) topics differing greatly from goal setting/action planning (e.g. learning goals of students, surgical interventions, coping with advanced directives) (Fig 1 Study selection.).

At each stage, we divided the articles into relevant (R), irrelevant (I) and doubtful (D). In order to validate the selection procedure, the inclusion and exclusion criteria were checked for consistency by the two reviewers.

### Charting the data

A data-charting form was jointly developed by two reviewers to determine which variables to extract. The two reviewers independently charted the data, discussed the results and continuously updated the data-charting form in an iterative process.

This data-charting form contained descriptive variables (year of publication, study design, setting, target group) and information about the goal setting/action planning. We extracted information about the way authors defined goal setting and/or action planning and information about the way they intended to apply goal setting and/or action planning. For studies aiming to evaluate the effectiveness or feasibility of goal setting and/or action planning interventions, we also reviewed data on fidelity (i.e. the degree to which an intervention is delivered as intended).

### Collating, summarizing and reporting the results

Focusing on disentangling goal setting and action planning, we applied a qualitative content analysis approach [26]. The analysis resulted in (1) an overview of goal setting phases (defined as steps of the goal setting/action planning process); (2) an overview of components (defined as activities performed in each of the goal setting phases) and (3) an overview of strategies (defined as techniques to put components into practice). The analysis also resulted in a description of the targets of goal setting/action planning, the theoretical underpinning and the mode of delivery of goal setting and/or action planning.

## Results

### Study characteristics

The search resulted in 9115 hits. After screening the titles, abstracts and full texts and correcting for duplicates, 58 articles met the inclusion criteria (see Fig 1 Study Selection.). Of the included articles, 46 (79%) were published in or after 2006, and most originated from the USA (n = 28, 48%) or Europe (n = 21, 36%). Most of the articles were RCTs (n = 25, 43%), pre-posttest trials (n = 13, 22%) or non-randomized controlled trials (n = 3, 5%). None of those intervention trials reported to have examined intervention fidelity. Six articles (10%) presented qualitative studies (e.g. use of a goal setting instrument to elicit patients’ goals, in order to study the content of these goals) and five articles (9%) described the development of an intervention (intervention development study) using mixed methods. The selection also included three case studies (5%), two study protocols (3%) and one literature study (2%). Fifty-four studies (93%) applied goal setting/action planning to patients suffering from one specific disease, mostly to those suffering from diabetes (n = 16, 28%) or neurological diseases (n = 10, 17%). Articles encompassed a wide range of settings (mostly primary care (n = 24, 41%), the community (n = 12, 21%) and the outpatient departments (n = 8, 14%)) (See Table 2 Summary of Study Characteristics.) (See Table 3 Study Characteristics.)

**Table 2:** Summary of study characteristics.

Characteristic	Categories for each characteristic	Number of studies (n = 58)
**Date of publication**	Before 1990 (1978)	1
	1991–1995	0
	1996–2000	2
	2001–2005	9
	2006–2010	19
	2011–2015	27
**Place of publication**	USA	28
	Europe	21
	Canada	3
	Australia	2
	Other	4
**Study design**	RCT	25
	Pre-posttest design	13
	Non-randomized controlled trial	3
	Qualitative design	6
	Intervention development study (using mixed methods)	5
	Case study	3
	Study protocol	2
	Literature study	1
**Target group**	Diabetes	16
	Neurological diseases	10
	Cardiovascular diseases	7
	Any chronic condition	4
	Mental illness	4
	Pain	3
	Rheumatoid arthritis	2
	Geriatric patients	2
	Asthma	2
	Cancer	2
	Other	6
**Setting**	Primary care	24
	Community	12
	Outpatient department	9
	Rehabilitation	5
	Hospital	3
	Residential care setting	3
	Pharmaceutical setting	1
	Other settings	1

**Table 3:** Study characteristics.

Author (year)	Study design	Research question	Patients	Professionals	Setting	Country
Arbour-Nicitopoulus et al. (2003)	RCT	To examine the effects of action and coping planning on physical activity and self-efficacy.	Spinal cord injury	Researcher	Community	Canada
Bacelar de Araujo Lourenco et al. (2013)	RCT	To assess the effect of action and coping planning on the adherence to drug treatment.	Coronary artery disease	researcher	Outpatient clinic	Brazil
Bearon et al. (2000)	Intervention Development Study	To describe the development and testing of the Personal Functional Goals (PFG) Checklist/Interview.	Geriatric patients	researcher	Primary care	USA
Becker et al. (2009)	RCT	To examine goal achievement over an 8-month period in women with fibromyalgia.	Fibromyalgia	nurse practitioner	Community	USA
Briggs-Early et al. (2009)	Qualitative design	To examine Latinos’ dietary behaviors used to achieve self-management goals.	Diabetes	nurse/ dietitian	Primary care	USA
Buechi et al. (2010)	Case study	To describe the implementation of Pictorial Representation of Illness and Self-measure (PRISM).	Psychiatric patients	psychotherapist	Psychiatric hospital	Switzer-land
Calfas et al. (2002)	RCT	To evaluate Patient-centered Assessment and Counseling for Exercise plus nutrition (PACE +).	Overweight	family physician/ nurse practitioner	Primary care	USA
Cho (2013)	RCT	To examine the effect of Health Contract Intervention on self-care behavior and physiological indices.	Renal dialysis patients	researcher	Outpatient hospital	Korea
Christiansen et al. (2010)	RCT	To evaluate a concise method for encouraging exercising in and consolidating behavioral changes.	Chronic back pain	psychologist	Outpatient rehabilitation	Germany
Chunchu et al. (2012)	Non-randomized controlled trial	To evaluate a team approach using a HER-based patient centered care plan (PCCP).	Any chronic condition	physician/ medical assistant	Primary care	USA
Corser et al. (2007)	Pre- posttest	To test the feasibility of a brief shared decision making goal setting intervention.	Diabetes	Nurse	Primary care	USA
Coote & MacLeod (2012)	RCT	To evaluate Goal setting and Planning Skills (GAP) in its individual, self-help format.	Depression	No professional	Community	UK
Custer et al. (2013)	Pre-posttest	To test a self-reported strategy that would assist in choosing goals and rating goal achievement.	Any chronic condition	Occupational therapists	Outpatient rehabilitation	USA
Davis & White (2008)	Pre-posttest	To test a pain self-management intervention, the Goal Attainment Pain Management Program (GAPAP).	Rheumatoid arthritis	Nurse	Residential care	USA
DeWalt et al. (2009)	Pre-posttest	To evaluate the Living with Diabetes Guide and a brief counseling intervention to set and achieve goals.	Diabetes	Research assistant	Primary care	USA
Dickman et al. (2012)	Pre-posttest	To evaluate changes in self-managing behavior following Shared Medical Appointments (SMAs).	Diabetes and hypertension	Nurse practitioner/ physician/ diabetes educator/medical assistant	Primary care	USA
Doughty et al. (2008)	Pre-posttest	To evaluate the delivery of a series of workshops on mental health recovery.	Mental illness	Mental health professional/ patients	Primary care	New Zeeland
Estabrooks et al. (2005)	RCT	To determine the frequency and effectiveness of goal choices and test goal setting theory hypotheses.	Diabetes	Physician	Primary care	USA
Evans-Hudnall et al. (2014)	RCT	To pilot a self-care treatment adapted for underserved racial/ethnic minority groups.	Stroke	Health educator	Hospital and community	USA
Fuchs et al. (2011)	Non-randomized controlled trial	To examine the effectiveness of the MOVO-Lisa intervention.	Orthopedic patients	Psychologist, physiotherapist	Outpatient rehabilitation	Germany
Glasgow et al. (2011)	RCT	To calculate various indices of website engagement.	Diabetes	Internist	Primary care	USA
Glasgow et al. (1996)	RCT	To evaluate an office-based intervention focused on behavioral issues for dietary self-management.	Diabetes	Internist	Primary care	USA
Glasgow et al. (2002)	RCT	To evaluate the effects of adding follow up components to a dietary goal-setting intervention.	Diabetes	Physician	Primary care	USA
Harris & Eng (2004)	Qualitative design	To identify goal priorities using a client-centered assessment.	Stroke	Occupational therapist	Community	Canada
Hart (1978)	RCT	To evaluate the effectiveness of setting goals in behavioral terms while monitoring attainment to goals.	Mental health problems	Psychotherapist	Community	USA
Holtrop et al. (2006)	RCT	To examine the types and influences of self-selected health behavior goals.	Cardio-vascular patients	Trained health educator	Hospital	USA
Kjeken et al. (2014)	Study protocol	To evaluate goal attainment, health effects and the cost-effectiveness of a new rehabilitation program.	Rheumatoid arthritis	Rehabilitation team	Rehabilitation	Norway
Kroese et al. (2014)	Pre-posttest	To investigate long-term outcomes of a self-management intervention targeting proactive coping.	Diabetes	Instructed trainers	Primary care	Nether-lands
Luszczynska (2006)	Pre-posttest	To investigate relations between implementation intention, a planning strategy and physical activity.	Myocardial infarction	Researcher	Rehabilitation	Poland
Lyons et al. (2015)	Qualitative design	To identify goals and patterns of goal attainment and to understand what women were trying to achieve.	Cancer	Occupational therapist	Hospital	USA
Magar et al. (2005)	RCT	To evaluate an educational program.	Asthma	Family practice professional	Community	France
Mansson Lexell et al. (2014)	Pre-posttest	To assess self-perceived performance and satisfaction with performance of daily activities	Multiple sclerosis	Occupational therapist	Rehabilitation	Sweden
McConkey & Collins (2010)	Qualitative design	To examine a goal setting approach and to identify the variables that influenced goal achievement.	Learning disabilities	Researcher	Residential care setting	Northern Ireland
Morganstern et al. (2011)	Qualitative design	To evaluate the Quality of Life Appraisal Profile.	Cancer	Researcher	Primary care	USA
Mullis & Hay (2010)	Intervention development study	To develop a goal-based individualized assessment tool capable of defining meaningful change.	Low back pain	Researcher	Primary care	UK
Murphy & Boa (2012)	Case study	To describe the use of the ICF framework, alongside Talking Mats, to enable participation in in goal setting.	Communi-cation difficulties	Rehabilitation professional	Outpatient rehabilitation	UK
Naik et al. (2011)	RCT	To evaluate the effectiveness of 2 interventions on glycosylated hemoglobin (HbA1c) levels.	Diabetes	Physician	Primary care	USA
Nuovo et al. (2009)	Pre-posttest	To assess differences between women and men in developing an action plan.	Diabetes	Diabetes educator	Community	USA
O’Connor et al. (2008)	RCT	To conduct a self-management intervention focusing on professional led vs patient led goal setting.	Intermittent allergic rhinitis	Pharmacist or pharmacy assistant	Pharmacy	Australia
Pagels et al. (2015)	Pre-posttest	To evaluate the effects of a group-based, multidimensional and multidisciplinary support program.	Diabetes	Nurse	Outpatient clinic	Sweden
Power et al. (2011)	Case study	To describe the application of the ICF to communication assessment and goal setting.	Huntington disease	Researcher	Residential care setting	Australia
Schneider et al. (2011)	Non-randomized controlled trial	To examine perception of life coaching and person-centered planning for maintaining employment and managing chronic health issues.	Diabetes	Life coach	Community	USA
Schreurs et al. (2003)	Intervention development study	To describe the development of a intervention to enhance self-management.	Asthma, diabetes, heart failure	Nurse	Primary care	Nether-lands
Scobbie et al. (2010)	Literature study	To describe the development of a theory-based goal setting practice framework.	Neurological diseases	Rehabilitation team	Rehabilitation	UK
Sniehotta et al. (2005)	RCT	To examine two interventions addressing self-regulatory skills in their effects on physical exercise.	Coronary heart disease	No professional	Rehabilitation	Germany
Stuifbergen et al. (2003)	RCT	To examine the effectiveness of a wellness intervention on achieving health-related goals.	Multiple sclerosis	Nurse practitioner	Community	USA
Steurer-Stey et al. (2015)	RCT	To examine the addition of the Zurich Resource Management training to a patient education program.	Asthma	Health educator	Primary care	Switzer-land
Theunissen et al. (2003)	RCT	To study whether illness representations and action plans change when general practitioners are trained.	Hypertension	Physician	Primary care	Nether-lands
Thoolen et al. (2009)	RCT	To examine the effectiveness of a proactive intervention.	Diabetes	Nurse	Primary care	Nether-lands
Tielemans et al. (2014)	Study protocol	To describe the theoretical basis and the content of the treatment protocol for ‘Plan ahead!’	Stroke	Rehabilitation professionals	Outpatient rehabilitation	Nether-lands
Tomori et al. (2012)	Intervention development study	To develop and evaluate an IPad application to promote shared decision making in goal setting.	Any chronic conditions	Occupational therapist	Not specified	Japan
Toto et al. (2015)	Pre-posttest	To determine the feasibility of generating patient-centered goals.	Geriatric patients	Rehabilitation team	Primary care	USA
Tripicchio et al. (2009)	Pre- posttest	To examine if training professionals in the OPN (Ozer, Payton & Nelson) method improves goal setting.	Any chronic condition	Physical therapist/ occupational therapists	Primary care	USA
Voils et al. (2013)	RCT	To evaluate the effectiveness of a telephone-delivered, spouse-assisted lifestyle intervention.	Coronary heart disease	Nurse	Primary care	USA
Walker et al. (2009)	Qualitative design	To examine and describe goal setting among people who participated in the pilot testing of WebEase	Epilepsy	No professional	Community	USA
Wolever et al. (2010)	RCT	To evaluate the effectiveness of integrative health coaching.	Diabetes	Coach	Community	USA
Van der Wulp et al. (2012)	RCT	To investigate a self-management coaching intervention.	Diabetes	Expert patients	Primary care	Nether-lands
Zhang et al. (2015)	Qualitative design	To enhance the understanding of patient motivation for self-care through the examination of their goals	Cardio-vascular patients	Researcher	Outpatient setting	Canada

### Targets of goal setting/action planning

With regard to the targets of goals and action plans, the articles showed great variation. Thirty-five studies (60%) aimed to support patients in setting goals or formulating action plans for one or more lifestyle behaviors [12, 13, 27–59]. Goal setting and action planning were mostly intended to improve physical activity (n = 21, 36%), healthy eating (n = 17, 29%), and/or medication adherence (n = 13, 22%). In these articles, the targets of goals/action plans had been predefined. Other articles (n = 9, 16%) focused more on everyday activities and participation [60–68] or on patients’ quality of life and well-being (n = 4, 7%) [69–72]. Nine of the articles (16%) did not describe in detail the goals they focused on, but reported that patients were asked to set health-related goals or goals for rehabilitation [14, 73–80]. One article focused on the development of an action plan for managing deterioration of mental well-being for people suffering from a mental illness [81].

### Theoretical framework

Nearly half of the articles (n = 27, 47%) reported a theoretical framework for goal setting/action planning. The most commonly used theoretical frameworks to underpin the use of goal setting or action planning as a method to change a person’s behavior were behavior change theories. The Social Cognitive Theory [82], in which self-efficacy is described as the key concept for setting goals [83], was mentioned in 14 articles (24%). Other behavioral change theories that were referred to were: Self-Regulation Theory [84] (n = 5, 9%), Proactive Coping Theory [85] (n = 4, 7%), Health Action Process Approach [86] (n = 3, 5%), Self-Determination Theory [87] (n = 1, 2%) and the Theory of Planned Behavior [88] (n = 1, 2%). These theories were reported to be used because of their focus on overcoming the intention-behavior gap and on planning behavior. The theory of Locke & Latham [89] was used in four articles (7%) to underpin the importance of specificity and difficulty of goals [14, 29, 37, 52]. One article reported the use of the 5 A´s framework [30]. This framework (Assess, Advise, Agree, Assist, Arrange) is a process model for self-management support that puts goal setting and action planning at the center of self-management support [8]. Although theories were thus mentioned in nearly half of the articles (n = 27, 47%), fifteen articles (26%) offered minimal or no explanation of the relation between theory and the development, content and format of goal setting and action planning.

### Goal setting phases and components

The five main goal setting phases identified were (1) preparation, (2) formulation of goals, (3) formulation of action plan, (4) coping planning, and (5) follow-up (for definitions of all phases see Table 4 Phases and Components.). Nineteen of the articles (33%) included all phases [12–14, 27–37, 69–71, 73, 74]. Different components were identified for the preparation, coping planning and follow-up phases (see Table 4 Phases and Components.).

**Table 4:** Phases and components.

Phase	Definition	Component
Preparation	Patients engage in activities prior to setting goals and making action plans (independently or with support from a professional).	Patient education (about the disease or relevant disease-related behaviors/factors)
		Patient reflection (on current behavior and attitudes)
		Identification of topics for setting goals
Formulation of goals	Patients’ goals are made explicit and are written down (independently or with support from a professional).	
Formulation of action plan	Patients’ action plans are made explicit and are written down (independently or with support from a professional).	
Coping planning	Patients explicitly formulate plans that describe how potential barriers to carrying out an action plan could be overcome (independently or with support from a professional).	Identification of barriers to carrying out the action plan
		Identification of facilitators for carrying out the action plan
		Assessment of confidence about carrying out the action plan
		Formulation of strategies to overcome barriers
Follow-up	Patients actively work on achieving their goals and/or are supported in working on their goals and carrying out their action plans.	Patients’ self-monitoring of progress towards goal achievement
		Support for the patients
		Evaluation of progress or achievement

The preparation phase was described in 49 of the 58 included articles (85%), and three components were identified: (1) patient education (about the disease or relevant disease-related behaviors/factors) (n = 22, 38%); (2) patient reflection (on current behaviors or attitudes) (n = 24, 41%); and (3) identification of topics for setting goals (any kind of topic or problem the patient wants to set a goal for) (n = 15, 26%). The formulation of goals was also reported in 49 articles (85%). The 9 articles (16%) that omitted the goal formulation phase incorporated the formulation of an action plan for a goal that was preset and identical for all participants [38–45, 81] (e.g. to increase physical activity). The formulation of an action plan was discussed in 38 articles (66%). The 20 articles (44%) that did not include the formulation of an action plan encouraged patients to set short-term behavioral goals, which were formulated as actions (e.g. feasible and measureable behavior goals for a lifestyle behavior). Thirty articles (52%) incorporated the phase of coping planning. Within this phase, our analysis revealed four components: (1) identification of barriers (factors that might impede goal achievement) (n = 24, 41%); (2) identification of facilitators (factors that might enhance goal achievement) (n = 6, 10%); (3) assessment of confidence (patient’s confidence about carrying out the action plan) (n = 7, 12%); and (4) formulation of strategies to overcome barriers (plans to overcome problems while working on goals) (n = 21, 36%). Forty-two articles (72%) mentioned a follow-up phase. In the follow-up phase, three components were identified: (1) patient self-monitoring (monitoring of progress in goal achievement by the patient) (n = 17, 29%); (2) support (practical or emotional support) for the patient (n = 17, 29%); and (3) evaluation of progress or achievement (interim evaluation of the progress and/or evaluation of goal achievement) (n = 25, 43%) (See S1 Table Overview of Phases and Components for each included Article).

### Strategies

This section describes strategies that were mentioned in the articles for the different phases of the goal setting process. Strategies were defined as practical techniques to put a phase or component into practice (See S2 Table Overview of Mode of Delivery, Phases, Components and Strategies for each included Article).

#### Strategies for preparation

In the preparation phase, patients were often educated by means of written or visual materials (like workbooks, brochure, video’s or a website) (n = 13, 22%) [12, 30, 34, 36, 37, 46–50, 69, 74, 75] and/or in group meetings (n = 7, 12%) [42, 46, 47, 51–53, 74]. Patient reflection or self-assessment of their current status or behavior was usually done independently by the patients, without the help of a professional, by means of questionnaires (on paper or on a computer) (n = 5, 9%) [27, 29, 33, 54, 55] or self-care logs/diaries (n = 4, 7%) [28, 30, 37, 42]. Bearon et al. (2000) used ladder rating as a visual technique to reflect on the patient’s present status in acht self-selected functional areas (60). Five other articles (9%) investigated techniques for patient reflection on positive and negative aspects of changing behavior [27, 31, 40, 45, 90]. Three of these articles (5%) used mental contrasting or mental simulation as a strategy (for more explanation see S2 Table) [40, 45, 90]. Six articles (10%) discussed the exploration of patients’ overall perspectives on life and health [56, 60, 70, 72, 73, 76]. Patients were asked to reflect on their wishes in life, their hopes and fears or values for health, using in-depth interviews [60, 73, 76] or instruments like the Brief Quality of Life Appraisal profile [70], the Personal Value Care Sort [72] or the Wheel of Health [56] (for more explanation see S2 Table). Exchanging experiences with a group of peer patients was reported in four articles (7%) [54, 74, 81, 90]. Thirteen other articles (22%) focused on patients’ problems in everyday life and described individual interviews to identify and analyze these problems [34, 43, 57, 61–65, 71, 77–79]. Three of these articles (5%) used the Canadian Occupational Performance Measurement (COPM), a standardized outcome measure designed to capture a patient’s self-perception of performance in everyday living over time [61, 62, 65] (for more explanation see S2 Table). In addition, three articles (5%) used motivational interviewing to identify topics for discussion [57, 71, 80] and one article described the use of the visual instrument Talking Mats [63](for more explanation see S2 Table).

#### Strategies for formulation of goals

With regard to strategies for goal formulation, 16 articles (26%) focused on strategies for goal formulation using established criteria for effective goal setting [12–14, 32, 34, 35, 37, 48–51, 56, 58, 66, 71, 78]. In these articles, patients were stimulated to set specific, achievable, measurable, realistic, time-bound and behavioral goals (SMART criteria). However, the SMART criteria were only explicitly mentioned in three articles (5%) [37, 49, 71]. Scobbie et al. (2011) added that goals needed to be challenging for the patient [14]. Two other articles (3%) described encouraging patients to focus on long-term goals, often concerning their participation or life goals [56, 66]. Documenting patients’ goals was described in 19 articles (33%). In 11 of these articles (19%), goals were written down for the patient in records, cards or worksheets [27–29, 33, 55, 64, 66, 67, 75, 76, 78]. Cho et al (2013) and Tomori et al. (2012) used the method of contracting to document goals. Goals were written in the form of a contract, which was signed by both the professional and the patient [28, 67]. Six articles (10%) used patient workbooks or manuals in which patients could write down their goals [12, 13, 53, 69, 74, 80]. In five articles (9%) this was supported by group meetings or individual meetings with a professional [12, 13, 53, 74, 80]. Another reported strategy was the use of checklists for goals (n = 5, 9%) [32, 55, 59, 60, 68]. Checklists for goals, like the Personal Goals Checklist [60] or the Goals for Occupational Therapy List [68] (for more explanation see S2 Table) were used to encourage patients to select items from a list of pre-formulated goals. Five of the included articles (9%) described the use of the Goal Attainment Scaling (GAS) instrument as a strategy to set goals. GAS is a method that supports patients to set specific goals and to score the extent to which goals are achieved (for more explanation see S2 Table) [47, 51, 65, 78, 79].

#### Strategies for formulation of an action planning

Eight articles (14%) also described characteristics for action plans, four of them (7%) mentioning the formulation of implementation intentions as a strategy [31, 39–41]. Implementation intentions include the three action plan characteristics of indicating when, where and how the action is to be carried out. Four other articles (7%) applied the same criteria to patients’ action plans, but did not explicitly mention the implementation intention strategy [14, 33, 38, 44]. The documentation of action plans was reported in 12 articles (21%) [29, 33, 35, 36, 38, 41, 43, 44, 50, 54, 67, 78]. Nuovo et al. (2009) used a decision wheel to explore topics to talk about and to produce an action plan, which was then written on the back of the wheel [43]. This action plan incorporated the following components: When will I do it? Where will I do it? How often will I do it? What might get in the way? What can I do about it? [43] (for more explanation see S2 Table). Other strategies that were described included discussing and rating the level of difficulty of actions [70], discussing action plans with peers [52], and mental contrasting as a method to imagine possible actions [45] (for more explanation see S2 Table). Voils et al. (2013) involved spouses by developing a specific behavioral plan for spouses to support the patients’ goal achievement [36]. Doughty et al. (2008) described the use of a personal crisis plan for a possible deterioration of mental well-being for patients suffering from mental illnesses [81] (for more explanation see S2 Table).

#### Strategies for coping planning

Nine articles (16%) described strategies used for coping planning. In contrast to goals and action plans, coping plans were only documented in five articles (9%). If documented, they were always written down together with the action plans [29, 35, 38, 43, 44]. Four articles (7%) mentioned the use of motivational interviewing and the use of cognitive behavioral principles of problem solving as strategies to set coping plans [30, 40, 51, 55] (for more explanation see S2 Table).

#### Strategies for follow-up

Patients’ self-monitoring was almost always done using logbooks or diaries (n = 8, 14%) [13, 31, 34, 38, 44, 47, 50, 71]. Support for the patient (i.e. encouragement of patients by professionals and/or peer(s) to keep working on goals or action plans) was done via telephone or e-mail in six articles (10%) [27, 32, 39, 53, 55, 80]. In three articles (5%), support was provided in group sessions [32, 39, 52]. Kroese et al. (2014) described an extensive phase of goal reinforcement by means of three ‘booster sessions’ to enhance the attainability of goals and to discuss patient’s higher-order overarching goals [58] (for more explanation see S2 Table). Six articles (10%) used telephone calls to review progress and update goals or actions [12, 29, 31, 36, 38, 47]. In four articles (7%), review of progress was done within individual or group meetings [43, 50, 75, 76]. Five articles described the use of the GAS instrument to measure goal attainment (9%) [47, 51, 53, 78, 79] (for more explanation see S2 Table). Two articles (3%) involved measuring satisfaction about goal attainment by using the COPM [62] or Goal Satisfaction Rating [68] (for more explanation see S2 Table). Hart (1978) reported using an interview with a person from the patient’s social environment who was significant to the patient’s goal, as external validation of the patient’s self-reporting [78]. One article specifically emphasized patients’ future goal planning by means of follow-up calls [69]. The follow-up calls were intended to stimulate the patients to continue goal setting [69].

### Mode of delivery

Goal setting phases, components and strategies varied significantly in terms of their duration, ranging from a single individual goal setting/action planning session to several meetings distributed over a year. The number of meetings with patients ranged from one to nine, and in 40 articles (69%), goal setting/action planning was applied at individual level or incorporated a mixture of individual and group sessions. Two articles (3%) presented goal setting/action planning as part of the usual/routine long-term care and therefore did not report the number of meetings [14, 73]. Forty-eight articles (83%) described face-to-face goal setting/action planning by a healthcare professional or researcher. In two articles (3%), the goal setting/action planning was supported by other (expert) patients, either only by expert patients [57] or by expert patients together with a healthcare professional [81]. Four articles (7%) described goal setting/action planning being provided by telephone calls and/or e-mail [34, 36, 48, 56]. In four other articles (7%), patients were taught to set their goals without the help of a professional, using a manual [44, 69] or a website [32, 37] (See S2 Table).

## Discussion

This scoping review aimed to disentangle how goal setting and action planning are defined within the context of the patient self-management literature. We found 58 articles reporting on phases, components and strategies for goal setting/action planning, which differed greatly in terms of their duration (from a single goal setting session to several meetings distributed over one year). The analysis resulted in an overview of five goal setting phases (preparation, formulation of goals, formulation of action plan, coping planning and follow-up). Although the goal-setting phases we found are in accordance with commonly used definitions and frameworks for goal setting [8, 10, 21], the majority of articles did not include all phases of the goal setting process. As most articles did not give any explanation for the inclusion of phases (neither from a theoretical nor from other viewpoints), the rationale for their use or non-use remains unclear. In addition, we have created an overview of different components within the goal setting phases and an overview of practical strategies to put components into practice. Interestingly, the largest numbers of strategies were found for the phase prior to formulating goals and action plans, and for the follow-up phase. We found few articles focusing on the communication or discussions about goals/action plans or on possibilities to tailor the goal setting/action planning to the individual patient. This is in line with our finding that goal setting and action planning mostly concentrated on a single disease and focused on the improvement of one or more predefined lifestyle behaviors. Although half of the articles reported a theoretical framework for goal setting/action planning, the relation between the theory and the definition, content and format of goal setting and action planning was usually not clearly stated.

The majority of the articles included in this review (n = 35, 60.3%) focused on lifestyle improvement (e.g. increasing physical activity or improving diet). Patients were encouraged to set their own goals and action plans within the lifestyle domain, but the overall nature of their goals was predefined. Moreover, most of the goal setting strategies were designed for people with one specific chronic condition and excluded people with different conditions or suffering from multi-morbidity. In addition, few articles started the goal setting process with an exploration of patients’ values, allowing them to set goals that differ from the predefined nature of goals. Self-management support models highlight the importance of starting self-management support with an exploration of the patients’ values and beliefs [8, 91]. Patients with chronic conditions want to be listened to and are more motivated to work on their self-management when their individual values, needs and preferences are taken into account [92, 93]. Focusing self-management programs on one single disease might carry the risk of narrowing self-management to complying with medical instructions and lifestyle regulations, without much attention for the individual patient’s values [94]. This contrasts with the holistic character of the definition of self-management, which highlights the equal importance of social self-management (i.e. adjusting behavior to prevent negative outcomes and maintaining or adjusting life roles) and emotional self-management (i.e. dealing with emotional responses to a chronic condition and coping with discomfort and disability) [7]. In addition, the interrelations between patients’ different goals (e.g. that dealing with medical symptoms is frequently related to adjusting social roles) might be disregarded [95–97]. Although self-management programs exist for different chronic conditions [98], more research into self-management goal setting and action planning, including patients with different chronic conditions or people with multi-morbidity, might be useful, allowing for the integration of the patients’ values and setting self-management goals at different levels.

In line with this, we also found that few articles elaborated on strategies for tailoring goal setting/action planning to the individual patient’s readiness to change. Literature about self-management and goal setting emphasizes that chronically ill patients often go through an adaptation process, in which they learn to adapt to their condition and change their behavior accordingly [99]. Patients in different phases of their behavior change process might require different approaches for self-management goal setting and action-planning as regards forms of communication and the intensity of support [100, 101]. Earlier research has shown that self-management programs are frequently designed for individuals who are ready to change [101]. These programs might only reach a minority of those in need [101]. More research into the way self-management goal setting and action planning can be tailored to the individual patient’s adaptation process might be useful.

With regard to the strategies identified in this review, it is worth noting that a large variety of articles focused on characteristics of goals or action plans or on instruments facilitating goal setting, like the GAS [102] or the COPM [103]. Few articles focused on discussion and communication about goals, action plans or coping plans, or on strategies for the decision making process. Although principles of motivational interviewing [104] and cognitive behavior theory principles [105] were mentioned as communication strategies in some articles, they often failed to describe how these strategies were used to decide on goals, action plans or coping plans. This is surprising, since goal-setting is frequently regarded as a complex interactional activity, in which professionals and patients can face many communication problems [17]. Earlier research has shown that patients and professionals often find it difficult to make shared decisions about goals or actions [106–108]. Difficulties often relate to dealing with conflicting perspectives and priorities, ensuring patient participation, exploring the patient’s perspective and agreeing on goals and plans [17, 106, 107]. The findings of the present review indicate that uncertainty remains about the communication practices that are applied in setting goals, action plans and coping plans.

Our review has several strengths. First, we used a comprehensive search strategy across multiple databases with no date restrictions, minimizing the risk of having missed scientific articles about self-management goal setting/action planning. In addition, the process of including articles and extracting charting data was done by two researchers, to enhance the trustworthiness [26]. However, the results of the review may also have been subject to certain limitations. First, in our search we used a combination of keywords for the different concepts, but self-management, goal setting and action planning are broad concepts whose terminology varies greatly. It is possible that we have missed articles that used other terms with similar meanings. On the other hand, we checked reference lists to minimize this potential shortcoming. Second, we limited our search to databases of peer-reviewed, scientific articles. Books and grey literature were not included. As a result, we may have missed some publications describing self-management goal setting/action planning. However, we were especially interested in ways in which goal setting and action planning are defined in the scientific literature, and we did not expect to find this within books and grey literature. In future research it would be worthwhile to review goal setting/action planning fidelity, in order to explore barriers and facilitators for implementation.

Another problem we faced was that scientific publications are frequently restricted in terms of words and space, so most of the articles included, especially those on intervention trials, lacked a comprehensive description of their intervention. This restriction in the number of words might also be responsible for the minimal descriptions of the relation between the theoretical framework used and the definitions of goal setting and action planning. It is therefore possible that our overview of phases, components and strategies is incomplete and also that more authors than we found used a theoretical basis for their definition of goal setting/action planning. We attempted to minimize this risk by checking the references for other sources providing more detailed descriptions. In future studies, it might be worthwhile to actively approach the authors of the included studies for additional information.

### Conclusion and implications

This scoping review assessed how self-management goal setting and action planning are defined. Although we focused on goal setting/action planning as described in the scientific literature, we think that our results might also encourage health care professionals to become more aware of:

- the entire goal setting process, especially of the importance of the phase prior to setting goals and action plans and the follow up-phase;
- possibilities to support patients in preparing for goal setting and working on goals by applying strategies focusing on patients’ self-reflection (e.g. logbooks, questions about values for health, visual methods);
- possibilities to support patients (and professionals) in identifying and formulating goals/action plans using certain instruments (e.g. Talking mats, GAS, COPM).

This scoping review also resulted in some implications for research. More research might be useful to investigate:

- goal setting and action planning for people suffering from different conditions or multi-morbidity;
- strategies for tailoring self-management goal setting and action planning to the patient’s readiness to change;
- communication strategies for goal setting and action planning that help patients and professionals overcome possible interactional difficulties (such as patient participation and agreement on goals).
- effectiveness of goal setting/action planning on patients’ behavior and medical outcomes. Those studies should investigate intervention fidelity in order to gain a better understanding of how and why an intervention works.

## Supporting information

S1 Table Overview of phases and components for each article. (PDF)

S2 Table Overview of mode of delivery, phases, components and strategies for each article. (PDF)
