# Supplementary material for: Disentangling self-management goal setting and action planning: A scoping review
Source: PLoS One. 2017 Nov 27;12(11):e0188822. doi: 10.1371/journal.pone.0188822 (PMC5703565; doi:10.1371/journal.pone.0188822)
Supplement: S1 Table — (PDF) [file pone.0188822.s001.pdf]

S2 Table. Overview of Phases and Components for each Article

	**Phases**											
	Preparation			Formulation of goals	Formulation of action plan	Coping planning				Follow up		
	**Components**											
**Articles**	Patient education	Patient reflection	Identification of topics for setting goals	Formulation of goals	Formulation of action plan	Identification of barriers	Identification of facilitators	Assessment of confidence	Formulation of strategies to overcome barriers	Patient self-monitoring	Support for the patient	Evaluation of progress
Arbour-Nicitopoulus et al. (2003)					x	x			x	x		x
Bacelar de Araujo Lourenco et al. (2013)					x	x			x		x	
Bearon et al. (2000)				x						x		
Becker et al. (2009)	x			x					x			x
Briggs-Early et al. (2009)				x		x			x			
Buechi et al. (2010)			x	x								
Calfas et al. (2002)		x		x	x	x	x		x		x	
Cho (2013)		x		x	x	x	x				x	x
Christiansen et al. (2010)		x			x	x			x			
Chunchu et al. (2012)		x		x	x	x		x		x		x
Corser et al. (2007)	x			x							x	
Coote & MacLeod (2012)	x			x	x	x			x	x	x	
Custer et al. (2013)				x								x
Davis & White (2008)	x			x						x		x
DeWalt et al. (2007)	x			x	x	x		x	x			x
Dickman et al. (2012)		x		x	x							
Doughty et al. (2008)	x				x							
Estabrooks et al. (2005)		x		x	x	x			x			x
Evans-Hudnall et al. (2014)	x	x		x	x				x	x	x	
Fuchs et al. (2011)		x		x	x				x	x		x
Glasgow et al. (2012)	x			x	x	x			x	x	x	
Glasgow et al. (1996)		x		x	x			x			x	
Glasgow et al. (2002)		x		x		x					x	x
Harris & Eng (2004)		x	x	x								
Hart (1978)			x	x	x							x
Holtrop et al. (2006)	x			x							x	x
Kjeken et al. (2014)				x	x			x			x	
Kroese et al. (2014)				x	x	x			x	x	x	x
Luszczynska (2006)	x				x						x	
Lyons et al. (2015)	x		x	x	x	x			x	x		
Magar et al. (2005)	x				x					x		

	Phases											
	Preparation			Formulation of goals	Formulation of action plan	Coping planning				Follow up		
	**Components**											
**Articles**	Patient education	Patient reflection	Identification of topics for setting goals	Formulation of goals	Formulation of action plan	Identification of barriers	Identification of facilitators	Assessment of confidence	Formulation of strategies to overcome barriers	Patient self-monitoring	Support for the patient	Evaluation of progress
Mansson Lexell et al. (2014)		x	x	x								x
McConkey & Collins (2010)				x								x
Morganstern et al. (2011)		x		x	x		x					x
Mullis & Hay (2010)			x	x								
Murphy & Boa (2012)			x	x								
Naik et al. (2011)	x			x	x							x
Nuovo et al. (2009)	x		x		x	x		x	x			x
O'Connor et al. (2008)	x			x	x					x		
Pagels et al. (2015)	x	x		x							x	
Power et al. (2011)			x	x								
Schneider et al. (2011)			x	x	x	x	x		x	x		
Schreuers et al. (2003)	x	x		x	x					x		x
Scobbie et al. (2010)		x	x	x	x	x		x	x	x	x	x
Sniehotta et al. (2005)					x	x			x	x		
Stuifbergen et al. (2003)	x			x		x	x				x	x
Steuer-Stey et al. (2015)		x		x	x	x	x					x
Theunissen et al. (2003)		x			x							
Thoolen et al. (2009)		x		x	x	x			x	x		
Thielemans et al. (2014)	x	x		x	x	x			x			x
Tomori et al. (2012)			x	x	x							
Toto et al. (2015)			x	x								
Tripicchio et al. (2009)			x	x	x							
Voils et al. (2013)	x			x	x			x			x	x
Walker et al. (2009)	x	x		x	x	x			x			x
Wolever et al. (2010)	x	x		x	x							
Van der Wulp et al. (2012)		x	x	x		x						
Zhang et al. (2015)		x		x								
