# Supplementary material for: Disentangling self-management goal setting and action planning: A scoping review
Source: PLoS One. 2017 Nov 27;12(11):e0188822. doi: 10.1371/journal.pone.0188822 (PMC5703565; doi:10.1371/journal.pone.0188822)
Supplement: S2 Table — (PDF) [file pone.0188822.s002.pdf]

S3 Table. Overview of Mode of Delivery, Phases, Components and Strategies for each Article

	**Mode of delivery**	**Phases**	**Components**	**Strategies**
Arbour-Nicitopoulus et al. (2003)	1 individual meeting (20 – 30 min), 1 call, follow up: 10 weeks	Formulation of an action plan		Calendars for patients: Recording time, place, number of minutes and intensity of physical activity
		Coping planning	Identification of barriers	
			Formulation of strategies to overcome barriers	Recording on planning sheets for patients at home
		Follow-up	Patient self-monitoring	Logbooks (10 weeks)
			Evaluation of progress	Telephone call (in week 5)
Bacelar de Araujo Lourenco et al. (2013)	2 individual meetings, 2 calls, follow up: 2 months	Formulation of action plan		Implementation intentions for medication adherence: Action plan covering when, where and how
		Coping planning	Identification of barriers	
			Formulation of strategies to overcome barriers	
		Follow-up	Support for the patient	Telephone calls (after 15 and 45 days)
Bearon et al. (2000)	2 individual meetings (baseline and outcome interview)	Formulation of goals		Personal Functional Goals Checklist: Naming and prioritizing goals with a 45-item checklist of goals for physical/mental health, daily living, general well-being
		Follow-up	Patient self-monitoring	Open-ended questions to reflect on wishes, hopes, fears and worries for future; ladder rating to reflect on present status in 8 self-selected functional areas: Pointing to a rung on a picture of a ladder (top rung (10) represents the best possible)
Becker et al. (2009)	8 group sessions (weekly, 2 hours), 1 individual meeting, 6 calls (bimonthly, 3 months), follow up: 8 months	Preparation	Patient education	
		Formulation of goals		Setting 2– 4 specific, feasible and measureable goals (for physical activity, nutrition, stress management, relationship/intimacy, life style adjustment, health management); Goal Attainment Scaling (GAS): Use of a 5-point scale (Zero point: Expected outcome/ goal, +1: a somewhat better than expected outcome, +2: the most favorable outcome, -1: a less than expected outcome, -2: a least favorable outcome)
		Coping planning	Formulation of strategies to overcome barriers	Motivational interviewing
		Follow-up	Evaluation of progress	

	Mode of delivery	Phases	Components	Strategies
Briggs-Early et al. (2009)	1 individual meeting (40 min)	Formulation of goals		"Reach for a goal": List of suggested self-management areas (exercise, diet, medication, eye exam, quit smoking, self-monitor blood sugar, and daily foot care) including a picture for each associated word/phrase.
		Coping planning	Identification of barriers	Use of the term "road block" to talk about barriers and strategies
			Formulation of strategies to overcome barriers	
Buechi et al. (2010)	1 individual meeting (45 min)	Preparation	Identification of topics for goals	Open questions; use of Pictorial Representation of Illness and Self-Measure (PRISM): Visualization (by means of a white board and moveable discs) of problems and resources, setting problem goals and resource goals
		Formulation of goals	Formulation of goals	
Calfas et al. (2002)	1 individual meeting (2 – 5 min), 8 bimonthly mails or 3 10-min calls and mails every 6 weeks OR weekly 10-min calls and mails	Preparation	Patient reflection	Computer to assess current behavior and compare current behavior to guidelines (exercise and nutrition)
		Formulation of goals		
		Formulation of action plan		Developing an action plan for exercise and nutrition on the computer; printing the action plan
		Coping planning	Identification of barriers	
			Identification of facilitators	
			Formulation of strategies to overcome barriers	
		Follow-up	Support for the patient	Telephone calls and/or mails with behavioral suggestions and information
Cho (2013)	4 weekly group meetings (30 – 60 min)	Preparation	Patient reflection	Self-care log (fistula management, blood pressure, body weight management, exercise and eating); group meetings with focus on knowledge and self-efficacy
		Formulation of goals		Contracting: Goals for one week and goal achievement methods are written down on contract that is signed by patient and professional
		Formulation of action plan		
		Coping planning	Identification of barriers	
			Identification of facilitators	
		Follow-up	Support for the patient	Praise, encouragement and support during usual dialysis sessions
			Evaluation of progress	Self-care logs

	Mode of delivery	Phases	Components	Strategies
Christiansen et al. (2010)	2 individual meetings (30 min)	Preparation	Patient reflection	Rating of expectations of success and incentive value with a 11 point response scale; mental contrasting: Listing 4 positive aspects of desired behavior (exercising more) and 4 obstacles, elaboration and visualization of the positive aspects and obstacles
		Coping planning	Identification of barriers	Cognitive behavioral principles of problem solving
			Formulation of strategies to overcome barriers	
		Formulation of action plan		Implementation intention (Action plan covering when, where and how)
Chunchu et al. (2012)	Routine long-term chronic care	Preparation	Patient reflection	Routine consultations family physician: Identification of preferences and values through dialog, use of a Patient Centered Care Plan (PCCP) in the Electronic Health Record (HER) (3 sections: About Me, My Goals, My Progress)
		Formulation of goals		
		Formulation of action plan		
		Coping planning	Identification of barriers	
			Assessment of confidence	
		Follow-up	Evaluation of progress	
Corser et al. (2007)	2 group meetings (120 min), workbook for patients	Preparation	Patient education	Education about medical evidence and the concept of shared decision making; workbook for patients with checklist and tools to help them set goals and discuss goals with their physician (28 pages)
		Formulation of goals		
		Follow-up	Support	
Coote & MacLeod (2012)	Workbook for patients (5 weeks), 1 call (in week 2)	Preparation	Patient education	Goal Setting And Planning Skills Manual (46 pages): Focus on setting positive goals for well-being, including practical tasks and worksheets
		Formulation of goals		
		Formulation of action plan		
		Coping planning	Identification of barriers	
			Formulation of strategies to overcome barriers	
		Follow-up	Support for the patient	Telephone call (week 2): Support and identifying new goals
			Evaluation of progress	
Custer et al. (2013)	2 individual meetings (intake and follow up)	Formulation of goals		Goals for Occupational Therapy List (GOTL): 25 item checklist that allows patients to select 3 goals for occupational therapy
		Follow-up	Evaluation of progress	Goal Satisfaction Rating: For each goal, patients rate their goal achievement and satisfaction on a 5 point scale.

	Mode of delivery	Phases	Components	Strategies
Davis & White (2008)	3 group meetings (1 h), 2 individual meeting with 2 nurses, 3 calls, workbook for patients, duration: 4 months	Preparation	Patient education	Group meetings: Education about pain management and goal-setting, exercises and strategies for pain modulation and pain relief using medicine and physical measures; notebook for patients (with exercises pain management)
		Formulation of goals		Goal Attainment Scaling (GAS): Use of a 5-point scale (Zero point: Expected outcome/goal, +1: a somewhat better than expected outcome, +2: the most favorable outcome, -1: a less than expected outcome, -2: a least favorable outcome)
		Follow-up	Patient self-monitoring	Diary
			Evaluation of progress	Telephone calls, GAS: Use of the 5-point scale (see formulation of goals) to rate goal attainment
DeWalt et al. (2007)	1 individual meeting (15 min), 2 calls, workbook for patients	Preparation	Patient education	Guide “Living with Diabetes”: Education about diet, exercise, monitoring, blood sugar, foot exams, insulin and other medicines, each chapter of the guide ends with a section to set small achievable goals
		Formulation of goals		
		Formulation of action plan		Action plans should be behavioral and specific about how, when and where
		Coping planning	Assessment of confidence	Self-rating of confidence to carry out the action plan on a scale from 0 - 10
			Identification of barriers	Telephone calls (after 2 and 4 weeks)
			Formulation of strategies to overcome barriers	
		Follow-up	Evaluation of progress	
Dickman et al. (2012)	Shared medical appointments (SMA) (8 – 12 participants, 90 min)	Preparation	Patient reflection	Reflection on current behavior (regarding nutrition, exercise and blood glucose monitoring) by means of discussions during SMA and by means of a questionnaire
		Formulation of goals		“My Action Plan”: Pictorial standard plan to set goals and plan actions
		Formulation of action plan		
Doughty et al. (2008)	4 group meetings/ workshops (1 or 2 full days)	Preparation	Education	Small group discussions about and exchanging experience over: Recovery concepts, getting medical care and handling medication, tools to use in daily life to enhance wellness, identification of triggers and symptoms
		Formulation of action plan		Personal crisis plan: List of supporters (their roles and phone numbers), medication the person uses, symptoms that indicate the person needs their supporters and instructions that what supporters

	Mode of delivery	Phases	Components	Strategies
Estabrooks et al. (2005)	Computer session (30 – 45 min), 1 individual meeting (8 – 10 min), 1 call	Preparation	Patient reflection	Interactive computerized touch screen CD ROM session to self-asses current behavior and compare it to recommended guidelines (dietary intake and physical activity)
		Formulation of goals		Computer to set goals and plan actions, generation of a printed copy of the action plan for the patient
		Formulation of action plan		
		Coping planning	Identification of barriers	
			Formulation of strategies to overcome barriers	
		Follow-up	Evaluation of progress	Individual meeting, telephone call (2 weeks later)
Evans-Hudnall et al. (2014)	1 individual session (30 – 45 min), 2 calls, workbook for patients	Preparation	Patient education	Patient workbook: Education about signs and symptoms, risk factors, behavioral strategies, resources for assistance, dietary and exercise tracking forms.
			Patient reflection	Diary about diet, physical activity, medication adherence, tobacco use and alcohol use
		Formulation of goals		Individual meetings: Use of cognitive behavioral principles (cognitive restructuring, strategies for social support, stimulus control, stress management, relapse prevention), patient workbook
		Coping planning	Identification of barriers	
			Formulation of strategies to overcome barriers	
		Formulation of action plan	Formulation of action plan	
		Follow-up	Support	Telephone calls (biweekly over 4 weeks after discharge)
Fuchs et al. (2011)	2 group meetings (6 patients, 60 - 90 min), 1 individual interview (10 min), 1 call		Evaluation of progress	
		Formulation of goals		Asking participants for objectives and willingness to work on goals and checking self-concordance by asking participants whether the goal is really their own or merely an introjection of others
		Preparation	Patient reflection	Contemplation of actions: Balancing the pros and cons of actions and reflecting on self-efficacy beliefs; reflection on outcome experiences: Consciously notice the positive consequences of new behavior.
		Formulation of action plan	Formulation of action plan	Implementation intentions for exercise: Action plan covering when, where and how
		Coping planning	Formulation of strategies to overcome barriers	
		Follow-up	Patient self-monitoring	Recording behavior and sending it to the professional
			Evaluation of progress	Telephone call (5 weeks after discharge) Telephone call (5 weeks after discharge)

	Mode of delivery	Phases	Components	Strategies
Glasgow et al. (2012)	Individual use of a website, for one intervention group also: 2 follow up calls, 1 group session	Preparation	Patient education	Website “My Path to health Life”: Short video about controlling hemoglobin A1c, blood pressure, cholesterol, following advice regarding medication, adherences, exercise and food
		Formulation of goals		“My Path to health Life”: Selection of achievable goals from a set of preset goals to enhance self-efficacy in medication adherence, exercise and food choices
		Follow-up	Patient self-monitoring	“My Path to health Life”: Tracking behavior (at least weekly) or use of an interactive voice response phone system
			Support for the patient	Motivational messages (weekly): Feedback on success or struggles
		Formulation of action plan		“My Path to health Life”: Choosing from prescribed actions to create an action plan to meet goals (with option “write your own actions”)
		Coping planning	Identification of barriers	“My Path to health Life”: Choosing from prescribed barriers and strategies (with option “write your own”)
			Formulation of strategies to overcome barriers	
		Follow-up	Support for the patient	For one intervention group: Social support through group sessions, telephone calls (after 2 and 8 weeks)
Glasgow et al. (1996)	1 meeting with physician, 1 meeting with intervention staff member (20 min)	Preparation	Patient reflection	Computer: Self-assessment of fat intake patters and assessment of barriers to dietary self-care, printed one page feedback form with results and suggestions for problems to focus on
		Formulation of goals		Printed goal setting worksheet and self-help pamphlet with concrete goals based on assessment results and strategies to achieve goals
		Formulation of action plan	Formulation of action plan	
		Coping planning	Assessment of confidence	Use of a 100 point scale to rate confidence
		Follow-up	Support for the patient	(Interactive) take-home video (30 min) on most frequent barriers to health eating and strategies to overcome barriers
Glasgow et al. (2002)	3 individual meetings (+ 7 calls or community resources follow up)	Preparation	Patient reflection	Touchscreen computer: Self-assessment of dietary patterns, barriers to and support for dietary self-management and feedback on current behavior
		Formulation of goals		Suggestion from the physician for dietary fat reduction goals based on the participant’s eating patterns and degree of patient’s self-efficacy, hand out with goals
		Coping planning	Identification of barriers	Motivational interviewing
		Follow-up	Evaluation of progress	Follow-up visits (after 3 and 6 months): Additional goals for further dietary change with focus on vegetable and fruit intake; relapse prevention and maintenance strategies: 1) Telephone follow-up calls (7 calls, 15 – 20 min) or 2) Community resources follow up (3-ring binder of indexed community resources, 8 newsletters + goal setting for community nutrition support activities).
			Support for the patient	

	Mode of delivery	Phases	Components	Strategies
Harris & Eng (2004)	1 individual meeting	Preparation	Identification of topics for setting goals	Canadian Occupational Performance Measure (semi-structured interview):<br>Exploration of everyday activities that patient perceives difficult to perform (areas: Self-care, productivity and leisure), rating of importance of each activity, prioritizing activities (up to 5), rating the self-perceived performance and satisfaction of activities (Ratings on Visual Analogue Scale from 0 – 10)<br>Total score: Scores identified for each problem or task are added and then divided by the number of problems or tasks.
			Patient reflection	
		Formulation of goals		
Hart (1978)	Routine care: several consultations	Preparation	Identification of topics for setting goals	Behavioral Monitoring Progress Record: Record including problems areas,<br>4-weeks goals, weekly goals (Observable, definable, measureable), method of attainment (behavioral assignments)
		Formulation of goals		
		Formulation of action plan		
		Follow-up	Evaluation of progress	Goal Attainment Scaling (GAS): Use of a 5-point scale (Zero point: Expected outcome/goal, +1: a somewhat better than expected outcome, +2: the most favorable outcome, -1: a less than expected outcome, -2: the least favorable outcome); interview with a person from the patient's social environment to validate patient's self-report
Holtrop et al. (2006)	6 calls (15 – 30 min, after hospital discharge), patient workbook	Preparation	Patient education	Booklet for patients (25 page): Education about risks and advantages of changing behavior, identification of at least 1 current behavior (tobacco use, physical activity, healthy eating) and identification of weekly specific, measurable, realistic goals (goal tracking worksheets)
		Formulation of goals		
		Follow-up	Evaluation of progress	Telephone calls: Review progress, relapse prevention, social support, future goal planning
			Support for the patient	
Kjeken et al. (2014)	2 individual meetings, 4 calls (after 1 week, 1 month, 4 months and 5 months)	Formulation of goals		Motivational interviewing, cognitive behavioral principles; self-management booklet (2 chapters): Goal setting, motivation to change, positive self-talk, choosing what to pay attention to and worth remembering, topics of relevance for the first period after admission. Possible to answer open questions, make notes, small exercises rating scales (e.g. to rate self-efficacy), quotes goals etc. from other patients, pictures and poems + activity diary
		Formulation of action plan		
		Coping planning	Assessment of confidence	
		Follow-up	Support for the patient	Telephone calls
Kroese et al. (2014)	1 individual session and 4 group sessions, (6 - 8 participants) (3 months)	Formulation of goals		Individual and group meetings: Stimulation to set small concrete, attainable goals (focusing on eating behavior, physical activity, medical regimes, topic of own choice)
		Coping planning	Identification of barriers	
			Formulation of strategies to overcome barriers	
		Formulation of action plan		
		Follow-up	Patient self-monitoring	Keeping track of goals homework assignments<br>3 booster sessions (1, 3 and 4 months after the intervention)<br>"How" booster: Reinforcement of focus on small, concrete, attainable goals OR "Why" booster: Elaborating on the self-management goals in view of their higher-order overarching goals, answering the "why" question repeatedly.
			Evaluation of progress	
			Support for the patient	

	Mode of delivery	Phases	Components	Strategies
Luszczynska (2006)	1 individual meeting (10 – 15 min)	Preparation	Patient education	Instructions that plans should always include the when, where and how
		Formulation of action plan	Formulation of action plan	Implementation intentions written on a form (about physical activity
		Follow-up	Support for the patient	Supportive feedback
Lyons et al. (2015)	Telephone calls (6) (weekly), workbook	Preparation	Education	Workbook: Education about activity engagement and health, the benefits of regular exercise and stress management techniques
			Identification of topics for setting goals	Identification of what makes an activity challenging
		Formulation of goals		Goals need to behavioral, measurable, and achievable within the next 7 days
		Formulation of action plan		Plans detailing when, where and how the goal will be met
		Coping planning	Identification of barriers	Weighting of advantages and disadvantages of solutions
			Formulation of strategies to overcome barriers	
		Follow-up	Patient self-monitoring	Workbook
Magar et al. (2005)	2 group sessions (150 min)	Preparation	Patient education	Group sessions: Education about signs of attack, medication, identification and avoiding trigger factors
			Patient reflection	Diary for asthma symptoms
		Formulation of action plan	Formulation of action plan	Group sessions: Action plan for controlling asthma including avoiding triggers, responding to warning signs, adapting treatment. Plan is based on symptoms and expiratory flow ranges, using a color system (green signals good control, yellow signals asthma is getting worse, red signals medical alert)
Mansson Lexell et al. (2014)	2 individual interviews	Preparation	Identification of topics for setting goals	Canadian Occupational Performance Measure (semi-structured interview):<br>Exploration of everyday activities that patient perceives difficult to perform (areas: Self-care, productivity and leisure), rating of importance of each activity, prioritizing activities (up to 5), rating the self-perceived performance and satisfaction of activities (Ratings on Visual Analogue Scale from 0 – 10)<br>Total score: Scores identified for each problem or task are added and then divided by the number of problems or tasks.<br>Evaluation of goals by rating self-perceived performance and satisfaction again at discharge.
			Patient reflection	
		Formulation of goals		
		Follow-up	Evaluation of progress	

	Mode of delivery	Phases	Components	Strategies
McConkey & Collins (2010)	3 individual interviews (each 9 months apart, with presence of key worker)	Formulation of goals		Individual structured interviews (with presence of key worker): 3 goals related to doing things with people and being involved in the communities for the next 6 – 9 months; participants were encouraged to independently identify activities to set goals on, goals formulated as precisely and as detailed possible; Goal record Sheet: Recording goals
		Follow-up	Evaluation of progress	Individual structured interviews (with presence of key worker): Discussion of factors that assisted or hindered the achievement of goals, Goal record Sheet: Recording goals and comment on what had proved helpful
Morganstern et al. (2011)	2 interviews via phone (prior and after surgery)	Preparation	Patient reflection	Brief Quality of Life Appraisal Profile: Patients are asked to respond to seven probes, representing different motivational orientations: achievement, problem resolution, prevention or avoidance of problems, maintenance or keeping situations as they are known, acceptance of circumstance, disengagement from roles and responsibilities and reaching life events or milestones (Example: In order to have the most satisfying life possible, what are the main things that you want to accomplish?), For each probe, patients were asked to provide up to 3 current goals (complete the goal statement "I want to.."); discussion and rating of level of difficulty of actions to reach the goals, discussion and rating of need for support
		Formulation of goals		
		Formulation of action plan		
		Coping planning	Identification of facilitators	
		Follow-up	Evaluation of progress	Rate progress on a scale from 0 – 10
Mullis & Hay (2010)	1 individual interview	Preparation	Identification of topics for setting goals	Individual semi-structured interview: Problem areas restricted to problems directly attributed to back pain
		Formulation of goals		Individual semi structured interview: Specific goals, asking questions about what patient would like to do, their past, the most important areas and about the problems that have the greatest impact, prioritizing 3 goals, Goal Attainment Scaling (GAS): Use of a 5-point scale (Zero point: Expected outcome/goal, +1: a somewhat better than expected outcome, +2: the most favorable outcome, -1: a less than expected outcome, -2: the least favorable outcome)
Murphy & Boa (2012)	Not specified	Preparation	Identification of topics for setting goals	1) Choosing a topic that the participant wants to talk about in relation to a specific issue (e.g. activities, environment, relationship, self-care), 2) Choosing options to talk about specifically related to a chosen topic (e.g. activities playing card games), 3) Indication of general feelings about each topic and option (e.g. whether they are happy, unsure); Talking Mats: Low-priced textured mat and picture symbols with Velcro attached to the back so that they can be placed on the mat and moved around. 3 sets of "Picture Communication Symbols": Topics, options and visual scale for indicating feelings, ICF: The ICF wording (activities and participation) was adjusted and the nine ICF domains matched to symbols of Talking Mats. A final set of 180 symbols/options were used
		Formulation of goals		

	Mode of delivery	Phases	Components	Strategies
Naik et al. (2011)	4 group sessions (5 - 7 members, 60 min, every 3 weeks over a 3 month period), 10 min individual interaction after each group session	Preparation	Patient education	Group sessions: Didactic education about diabetes and common risk factors, problem-based group discussion, group discussion, consultation time with clinical, instruction of principles of goal setting theory (goals specificity, difficulty, and importance) and setting goals together with physician; workbook
		Formulation of goals		Group sessions: Setting goals focusing on diet and exercise changes, home monitoring and medication effects, communication with physicians about medications, sleep, pain, barriers to self-management
		Formulation of action plan		Discussion of action plans with peers and physicians
		Follow-up	Evaluation of progress	
Nuovo et al. (2009)	4 group sessions (120 min)	Preparation	Patient education	Group sessions: Education about disease process, treatment options, nutrition physical activity, medication, monitoring blood glucose, complications, personal strategies for health and behavior change in group sessions using PowerPoint, white board, flip charts.
			Identification of topics for setting goals	Decision wheel with the following topics: weight, monitoring, exercise, depression, smoking, alcohol, sodium, medication, stress, nutrition. First prompt: "What would you like to talk about today". Then answering questions on the back of the decision wheel "My experiment this week", "When I will do it", "Where I will do it", "How often I will do it", "What might get in the way", "What can I do about it"?
		Formulation of action plan		
		Coping planning	Identification of barriers	
			Formulation of strategies to overcome barriers	
			Assessment of confidence	Rating of estimation of success "How successful do you think you will be?"
		Follow-up	Evaluation of progress	Group sessions
O'Connor et al. (2008)	3 individual meetings (baseline, 1 week after baseline and 6 weeks after baseline	Preparation	Patient education	Brochure about AR and about identification and avoidance of triggers
		Formulation of goals		Counseling with pharmacist: Setting 1 – 2 goals related to AR that are personally relevant to the patient and formulating actions to achieve those goals; recording goals and actions on a Card ''My Treatment Goals''
		Formulation of action plan	Formulation of action plan	
		Follow-up	Patient self-monitoring	

	Mode of delivery	Phases	Components	Strategies
Pagels et al. (2015)	3 group sessions (3 consecutive days, 6 hours, 6 participants)	Preparation	Education	Written educational material about the disease, multidisciplinary oral presentations, group discussions, Practicing blood glucose controls, injections technique, Nordic walking and seated gymnastics; medical review: Consultation with nephrologist and diabetologist for medical review and adjustment of present medical prescriptions
			Patient reflection	Worksheets: Participants fill in their health situation with respect to disease related factors such as glucose control or smoking
		Formulation of goals		Group discussions and worksheets: Participants set and record short-term and long-term health goals (for health and healthy life style behavior), according to the SMART model (specific, measurable, attainable, relevant and time-bound)
		Follow-up	Support for the patient	Feedback on performance
Power et al. (2011)	1 interview with patient and caregiver	Preparation	Identification of topics for setting goals	Semi-structured interview (open ended questions and probes) with patient and caregiver: exploration of communication-related activities and participation, current and future issues, structured according to ICF framework (functioning, disabilities, activities, participation, personal and environmental factors and potential future communication issues), use of Rehabilitation Problem Solving Form (RPS): Form to document patient and family perspectives of problems and disabilities and professional's perspective on mediators relevant to target problem (adding for this study: section for patient and family opinions on environmental, personal factors, future issues)
		Formulation of goals		
Schneider et al. (2011)	Several coaching sessions distributed over one year	Preparation	Identification topics for setting goals	Use of open-ended questions; motivational interviewing
		Formulation of goals		SMART goals for work, health and personal goals: specific, measurable, attainable, realistic, and timed
		Formulation of action plan		
		Coping planning	Identification of barriers	
			Formulation of strategies to overcome barriers	
			Identification of support	
		Follow-up	Patient self-Monitoring	Secure online coaching tool to put in goals, to update progress and to communicate between sessions

	Mode of delivery	Phases	Components	Strategies
Schreuers et al. (2003)	5 group sessions (2 hours, 6 - 8 participants, 4 sessions biweekly, 1 session after 4 weeks), workbook	Preparation	Patient reflection	Individual intake with a nurse: Reflection of and assessment of self-management behavior and knowledge
			Patient education	Workbook: Education about disease related themes: Physical condition, prevention of exacerbation, coping with negative emotions, giving and seeking social support
		Formulation of goals		Group discussions about personal goals and action plans, advice from the group about specificity and attainability, workbook with worksheets for action plans
		Formulation of action plan		
		Follow-up	Patient self-monitoring	Written daily register
			Evaluation of progress	Subsequent group sessions
Scobbie et al. (2010)	Continuous process (in rehabilitation)	Preparation	Identification of topics for setting goals	Goal negotiation: Appraisal of current situation and identification of the main problems, techniques to increase motivation, self-efficacy and positive outcome expectations such as verbal persuasion, modelling, re-interpretation of symptoms, mastery experience
		Formulation of goals		Goals should be specific and challenging and achievable within rehabilitation, self-set or assigned
		Formulation of action plan		Action plans: when, where and how, can it be written down?
		Coping planning	Assessment of confidence	Self-report
			Identification of barriers	
			Formulation of strategies to overcome barriers	
			Support for the patient	Appraisal of performance, measuring of progress and giving feedback, continuous reflection: Possible to go back to negotiation, setting or planning
		Follow-up	Evaluation of progress	
			Patient self-monitoring	
Sniehotta et al. (2005)	Independent of professional (after discharge from rehabilitation)	Formulation of action plan		Planning booklet with 2 planning sheets:<br>Action planning sheet: Room for 3 action plans for physical activity (When, Where, How, With Whom) + Coping planning sheet: Recording possible obstacles and coping plans
		Coping planning	Identification of barriers	
			Formulation of strategies to overcome barriers	
		Follow-up	Patient self-monitoring	Personalized Weekly Diary: Six weekly diaries by mail, recording progress, rating self-efficacy to adherence to plan and modify plans if necessary

	Mode of delivery	Phases	Components	Strategies
Stuifbergen et al. (2003)	8 group sessions (weekly), 6 calls (biweekly calls over 3 month period)	Preparation	Patient education	Group sessions: Education about lifestyle adjustment, physical activity, stress management, nutrition, healthcare responsibilities
		Formulation of goals		Group sessions: Worksheets are filled in at the end of each session incorporating goals, barriers and resources; Goal Attainment Scaling (GAS) via telephone: Use of a 5-point scale (Zero point: Expected outcome/goal, +1: a somewhat better than expected outcome, +2: the most favorable outcome, -1: a less than expected outcome, -2: the least favorable outcome)
		Coping planning	Identification of barriers	
			Identification of facilitators	
		Follow-up	Evaluation of progress	Telephone calls: Evaluation of progress and assistance in finding ways to overcome barriers, techniques to enhance self-efficacy: verbal persuasion, recognizing performance accomplishments, relating information abbot successful lifestyle changes of patients
			Support for the patient	
Steuer-Stey et al. (2015)	2 group sessions (whole day), 2 refresher sessions (120 min after 4 and 12 weeks)	Preparation	Patient reflection	Activation of personal resources: Patients are shown a set of pictures eliciting positive feelings and asked which picture evokes positive somatic responses (e.g. pleasant body feeling)
		Formulation of goals		Action- oriented personal goals; discussion in small groups; writing down goals, actions and potential barriers.
		Formulation of action plan		
		Coping planning	Identification of barriers	
			Identification of facilitators	Embodiment techniques, development of individual goal-related embodiment
		Follow-up	Evaluation of progress	Follow-up sessions: Discussion of goal progress and revision, transfer into daily life
Theunissen et al. (2003)	1 consultation (2 different conditions that are compared)	Preparation	Patient reflection	Condition 1: Discussion of illness representation, focus on adherence to blood pressure management advise; cognitive illness representation (“how do you think…”), emotional illness representation (“how do you feel..”)
		Formulation of action plan		Condition 2: Discussion of action plans, focus on adherence to blood pressure management advise, use of mental contrasting (a tool to envision possibilities and develop plans for bringing those possibilities about, by using one’s imagination)
Thoolen et al. (2009)	2 individual sessions, 4 group sessions (120 min, over 12 weeks), workbook	Preparation	Patient reflection	Individual session and group session: Discussion of experience with diabetes
		Formulation of goals		Group sessions: Set small, concrete and attainable goals and actions for physical exercise, diet and medication
		Formulation of action plan		
		Coping planning	Identification of barriers	Mental simulation (to help patients become more proactive, helping them anticipate potential barriers and virtually try out their goals and action plans); workbook for patients
			Formulation of strategies to overcome barriers	
		Follow-up	Patient self-monitoring	Written daily register of goal-attainment over 2 weeks

	Mode of delivery	Phases	Components	Strategies
Thielemans et al. (2014)	6 group sessions (120 min, 4 – 8 participants, 6 weeks), 1 booster session (week 10)	Preparation	Patient reflection	Group sessions and workbook: Exchanging of experience with stroke, education about consequences of stroke, setting goals also non-stroke related
			Education	
		Formulation of goals		
		Coping planning	Identification of barriers	
			Formulation of strategies to overcome barriers	
		Formulation of action plan		
		Follow-up	Evaluation of progress	
Tomori et al. (2012)	Application iPad (used together by patient and therapist)	Preparation	Identification of topics for setting goals	Aid for decision making in Occupational Choice: Application for the iPad with 94 illustrations describing daily activities related to activities and participation in the ICF, incorporating an importance-urgency matrix: Selection of important occupations (up to 20), rating of degree of importance by patient and by therapist(on 4-point scale), selection of 5 occupations, prioritizing occupation, rating degree of satisfaction for each occupation
		Formulation of goals		Documentation: Print out of therapy plan (goals and actions), patient and therapist sign the plan
		Formulation of action plan		
Toto et al. (2015)	1 individual session	Preparation	Identification of topics for setting goals	Semi structured interview, using the Canadian Occupational Performance (COPM) Measure as a framework: Hour by hour review of participants typical daily routine. Areas: self-care, productivity, leisure; exploration of everyday activities participants needed, wanted to do and were expected to do, rating of importance of each activity, selection of 2 – 4 activities for goal setting, Goal Attainment Scaling (GAS): Use of a 5-point scale (Zero point: Expected outcome/goal, +1: a somewhat better than expected outcome, +2: the most favorable outcome, -1: a less than expected outcome, -2: the least favorable outcome)
		Formulation of goals		
Tripicchio et al. (2009)	Whole process of treatment planning	Preparation	Identification of topics for setting goals	Use of different questions: What are your concerns? What are your goals? What has been accomplished? What actions have resulted in successful outcomes?, for each question 3 process: Exploration, selection (prioritizing), specification; use of 5 levels of patient participation: open-ended questioning, multiple choice, confirmed choice, forced choice and prescription
		Formulation of goals		
		Formulation of action plan		

	Mode of delivery	Phases	Components	Strategies
Voils et al. (2013)	9 patient calls (monthly), 9 spouses calls (monthly)	Preparation	Patient education	Written information about hypercholesterolemia and self-management principles; information about strategies to support patient goal achievement for spouses
		Formulation of goals		Patient calls: Measurable behavioral goals and action plans, patients are allowed to choose one behavior to focus on, recording of goals and action plans on provided paper, goals focusing on diet, physical activity, patient-physician communication, medication adherence, rating confidence to achieve the goals (on 10 point scale)
		Formulation of action plan		
		Coping planning	Assessment of confidence	
		Follow-up	Evaluation of progress	Patient calls; spouses calls: Information about patient goal and development of a specific behavior plan to support patient goal achievement.
			Support for patient	
Walker et al. (2009)	WebEase (interactive web site)	Preparation	Patient reflection	Web Ease (Interactive website): Diary (recording symptoms, medication taking, stress level, sleep habits)
			Patient education	Web Ease : Learning modules, discussion board, fact sheets, quizzes
		Coping planning	Identification of barriers	Web Ease
			Formulation of strategies to overcome barriers	
		Formulation of goals		Goals for sleep, stress, medication; SMART method (Specific, Measurable, Attainable, Realistic, and Timely), Web Ease
		Formulation of action plan		Web Ease
		Follow-up	Evaluation of progress	
Wolever et al. (2010)	14 telephone calls (30 minutes, 9 weekly calls, 4 biweekly calls and 1 call after 1 month)	Preparation	Patient education	Binder of educational materials
			Patient reflection	Exploration of values and priorities, health management and satisfaction and areas of required support; wheel of health to explore values, priorities and set goals: Identification of areas in which patients feel less successful or satisfied and choosing areas to focus on (areas: taking medicines as prescribed, stress reduction & self-care, exercise & physical activity, community & relationships, nutrition, personal development); motivational interviewing; creating a vision of health by asking questions about the bigger picture of life and the relation of health and the purpose in life
		Formulation of goals		Long-term goals for health aligned with vision of health
		Formulation of action plan		Small and realistic

	Mode of delivery	Phases	Components	Strategies
Van der Wulp et al. (2012)	3 monthly home visits (1 hour),<br>3 calls (2 weeks after the visit),<br>possibility to contact expert patient at any time by phone or mail	Preparation	Patient reflection	Exploration of areas for lifestyle change, appointment of importance and feasibility of life style change, focus on increasing patients' self-efficacy, improving physical activity and dietary habits, motivational interviewing
			Identification of important topics	
		Formulation of goals		Feasible goals to work on the upcoming month
		Coping planning	Identification of barrier	
Zhang et al. (2015)	1 scheduled routine appointment	Preparation	Patient reflection	Rating of importance of values: Modified version of the Personal Value Card Sorting Task, 5 domains of values (symptom-relief, physical well-being, social relationships, autonomy, hedonism)
		Formulation of goals		Goal setting (top 5, based on importance of values), 20 goal cards (based on domains of values): Participants are asked to sort each of the 20 goal cards into 3 piles "very important", "somewhat important", "less important",<br>measuring patient' s perception of compatibility of goals set with prescribed self-care behaviors (daily weighing, limiting sodium/ fluids and physical activity): The Competing Goals Task: Moving a tab along a -10 (not at all compatible) to +10 (very compatible) horizontal scale to indicate the degree to which they judge a self-care regimen facilitated or impeded achievement of a top 5 goals.
